# Supplementary material for: Single Cell Transcriptomic and Chromatin Profiles Suggest Layer Vb Is the Only Layer With Shared Excitatory Cell Types in the Medial and Lateral Entorhinal Cortex
Source: Front Neural Circuits. 2022 Jan 26;15:806154. doi: 10.3389/fncir.2021.806154 (PMC8826650; doi:10.3389/fncir.2021.806154)
Supplement: Supplementary file 14 [file Data_Sheet_1.ZIP › 14/knownResults.html]

14 - Homer Known Motif Enrichment Results


# Homer Known Motif Enrichment Results (14)

Homer *de novo* Motif Results  
Gene Ontology Enrichment Results  
Known Motif Enrichment Results (txt file)  
Total Target Sequences = 6338, Total Background Sequences = 42725

|  |  |  |  |  |  |  |  |  |  |  |  |
| --- | --- | --- | --- | --- | --- | --- | --- | --- | --- | --- | --- |
| Rank | Motif | Name | P-value | log P-pvalue | q-value (Benjamini) | # Target Sequences with Motif | % of Targets Sequences with Motif | # Background Sequences with Motif | % of Background Sequences with Motif | Motif File | SVG |
| 1 | A G C T A C G T A C T G A T G C A G T C C G T A C T G A T A C G | NF1-halfsite(CTF)/LNCaP-NF1-ChIP-Seq(Unpublished)/Homer | 1e-176 | -4.054e+02 | 0.0000 | 2227.0 | 35.14% | 8485.2 | 19.86% | motif file (matrix) | svg |
| 2 | C T G A C T G A C A T G A T C G A G C T A T C G G A C T C A T G C T G A G T C A | Tbr1(T-box)/Cortex-Tbr1-ChIP-Seq(GSE71384)/Homer | 1e-121 | -2.797e+02 | 0.0000 | 2162.0 | 34.12% | 9105.4 | 21.31% | motif file (matrix) | svg |
| 3 | G C T A G A C T G A C T T G C A C G T A A G T C C G T A T A G C G A T C G A C T | Eomes(T-box)/H9-Eomes-ChIP-Seq(GSE26097)/Homer | 1e-97 | -2.236e+02 | 0.0000 | 2890.0 | 45.61% | 14060.7 | 32.91% | motif file (matrix) | svg |
| 4 | C T G A C T A G A C T G G C A T C T A G G C A T A T C G C T G A C G T A G T C A | Tbx21(T-box)/GM12878-TBX21-ChIP-Seq(Encode)/Homer | 1e-93 | -2.155e+02 | 0.0000 | 1515.0 | 23.91% | 6054.9 | 14.17% | motif file (matrix) | svg |
| 5 | A G C T C A T G G C A T G A T C T G C A C T A G G A T C A C G T | Tgif2(Homeobox)/mES-Tgif2-ChIP-Seq(GSE55404)/Homer | 1e-63 | -1.462e+02 | 0.0000 | 3581.0 | 56.51% | 19617.8 | 45.91% | motif file (matrix) | svg |
| 6 | C G T A C T A G C A T G A G C T A C T G C G A T A T C G C G T A G T C A G T C A | Tbet(T-box)/CD8-Tbet-ChIP-Seq(GSE33802)/Homer | 1e-62 | -1.450e+02 | 0.0000 | 1499.0 | 23.65% | 6636.3 | 15.53% | motif file (matrix) | svg |
| 7 | C T A G C T G A T C A G A T C G A G C T C A T G G A C T A G T C C T G A T G C A | Tbx6(T-box)/ESC-Tbx6-ChIP-Seq(GSE93524)/Homer | 1e-57 | -1.331e+02 | 0.0000 | 1289.0 | 20.34% | 5580.7 | 13.06% | motif file (matrix) | svg |
| 8 | C A T G A G T C G A C T C G T A C G A T G C A T G A C T G C A T C G A T C T A G C A T G T G A C | Mef2b(MADS)/HEK293-Mef2b.V5-ChIP-Seq(GSE67450)/Homer | 1e-46 | -1.064e+02 | 0.0000 | 1423.0 | 22.46% | 6654.1 | 15.57% | motif file (matrix) | svg |
| 9 | G A C T C T A G A T G C A G T C G T C A T A C G A T G C A T C G | HIC1(Zf)/Treg-ZBTB29-ChIP-Seq(GSE99889)/Homer | 1e-44 | -1.034e+02 | 0.0000 | 1787.0 | 28.20% | 8852.0 | 20.72% | motif file (matrix) | svg |
| 10 | C A T G A G T C A G C T C G T A C G A T C G A T G C A T G C A T C G A T C T G A C A T G T G A C | Mef2d(MADS)/Retina-Mef2d-ChIP-Seq(GSE61391)/Homer | 1e-44 | -1.023e+02 | 0.0000 | 446.0 | 7.04% | 1449.7 | 3.39% | motif file (matrix) | svg |
| 11 | A G T C G A C T C A G T A C T G C T A G T G A C G C T A A T G C G C A T A T C G C G A T A C T G G A T C G T A C G T C A C T G A | NF1(CTF)/LNCAP-NF1-ChIP-Seq(Unpublished)/Homer | 1e-44 | -1.021e+02 | 0.0000 | 412.0 | 6.50% | 1293.8 | 3.03% | motif file (matrix) | svg |
| 12 | G T A C G A C T C G T A C T G A T C G A C G T A G C T A C A G T C T G A T A C G | Mef2a(MADS)/HL1-Mef2a.biotin-ChIP-Seq(GSE21529)/Homer | 1e-43 | -9.917e+01 | 0.0000 | 813.0 | 12.83% | 3322.4 | 7.78% | motif file (matrix) | svg |
| 13 | T C A G G A C T C A G T C T G A A G C T C T A G G A C T T G C A C T G A A G T C | HLF(bZIP)/HSC-HLF.Flag-ChIP-Seq(GSE69817)/Homer | 1e-40 | -9.357e+01 | 0.0000 | 1412.0 | 22.28% | 6760.2 | 15.82% | motif file (matrix) | svg |
| 14 | T C G A A C G T A C G T C T G A G A T C T C A G G A C T G T C A C G T A A G C T G T C A C T A G A G C T A C G T T C G A | NFIL3(bZIP)/HepG2-NFIL3-ChIP-Seq(Encode)/Homer | 1e-39 | -9.176e+01 | 0.0000 | 1094.0 | 17.26% | 4949.3 | 11.58% | motif file (matrix) | svg |
| 15 | C T G A C T A G A T C G A G C T A C T G G A C T A G T C C T G A | Tbx5(T-box)/HL1-Tbx5.biotin-ChIP-Seq(GSE21529)/Homer | 1e-36 | -8.442e+01 | 0.0000 | 2966.0 | 46.80% | 16632.0 | 38.93% | motif file (matrix) | svg |
| 16 | A G T C A T C G C T A G A G C T G A C T C T A G A G T C A G T C G C T A C A G T T C A G T C A G G A T C C T G A T C G A G A T C | RFX(HTH)/K562-RFX3-ChIP-Seq(SRA012198)/Homer | 1e-36 | -8.401e+01 | 0.0000 | 142.0 | 2.24% | 264.3 | 0.62% | motif file (matrix) | svg |
| 17 | G A C T G C A T C T A G C G A T G A T C T C G A C A T G G A T C | Tgif1(Homeobox)/mES-Tgif1-ChIP-Seq(GSE55404)/Homer | 1e-36 | -8.380e+01 | 0.0000 | 3330.0 | 52.55% | 19055.2 | 44.60% | motif file (matrix) | svg |
| 18 | G T A C G A T C C A G T A G T C A G T C A G T C T G C A G A T C C T G A A T G C G T C A A C G T | WT1(Zf)/Kidney-WT1-ChIP-Seq(GSE90016)/Homer | 1e-35 | -8.265e+01 | 0.0000 | 475.0 | 7.50% | 1720.4 | 4.03% | motif file (matrix) | svg |
| 19 | G A C T C A G T A G C T C G A T A G T C G A T C A G T C C G T A A T G C T C A G | Rbpj1(?)/Panc1-Rbpj1-ChIP-Seq(GSE47459)/Homer | 1e-34 | -8.054e+01 | 0.0000 | 1614.0 | 25.47% | 8160.5 | 19.10% | motif file (matrix) | svg |
| 20 | C A G T T C A G G A T C A C T G A C G T C T A G A C T G A C T G G A C T C T A G | Egr1(Zf)/K562-Egr1-ChIP-Seq(GSE32465)/Homer | 1e-33 | -7.741e+01 | 0.0000 | 501.0 | 7.91% | 1891.0 | 4.43% | motif file (matrix) | svg |
| 21 | C T A G A G C T G A C T C A T G A G T C A G T C G T C A C A G T C T A G T C A G G T A C C T G A T C G A G A T C T G A C | Rfx2(HTH)/LoVo-RFX2-ChIP-Seq(GSE49402)/Homer | 1e-33 | -7.731e+01 | 0.0000 | 157.0 | 2.48% | 335.2 | 0.78% | motif file (matrix) | svg |
| 22 | C A T G G T A C G A C T G C T A C G T A C G T A C G T A G C T A G A C T C T G A T C A G G T A C | Mef2c(MADS)/GM12878-Mef2c-ChIP-Seq(GSE32465)/Homer | 1e-31 | -7.265e+01 | 0.0000 | 883.0 | 13.93% | 3993.7 | 9.35% | motif file (matrix) | svg |
| 23 | T C A G A G C T A C G T A C G T G T A C G A T C C G T A C T A G C A T G G T C A C G T A T C G A | STAT4(Stat)/CD4-Stat4-ChIP-Seq(GSE22104)/Homer | 1e-28 | -6.667e+01 | 0.0000 | 1335.0 | 21.07% | 6709.1 | 15.70% | motif file (matrix) | svg |
| 24 | C A T G C T A G A G C T G A C T C A T G A G T C G A T C G C T A C G A T C T A G T C A G G T A C C T G A T C G A | X-box(HTH)/NPC-H3K4me1-ChIP-Seq(GSE16256)/Homer | 1e-24 | -5.629e+01 | 0.0000 | 195.0 | 3.08% | 571.6 | 1.34% | motif file (matrix) | svg |
| 25 | A C T G T C A G A G C T G A C T C A T G A G T C A G T C G C T A C G A T C T A G T C A G G T A C C T G A T C G A | Rfx1(HTH)/NPC-H3K4me1-ChIP-Seq(GSE16256)/Homer | 1e-22 | -5.289e+01 | 0.0000 | 252.0 | 3.98% | 850.1 | 1.99% | motif file (matrix) | svg |
| 26 | A C G T C T A G C G T A A G T C G T A C A C G T A C G T A C G T G T C A G T A C T G A C G A C T | Nur77(NR)/K562-NR4A1-ChIP-Seq(GSE31363)/Homer | 1e-22 | -5.125e+01 | 0.0000 | 332.0 | 5.24% | 1253.5 | 2.93% | motif file (matrix) | svg |
| 27 | A G T C C G A T A C T G A T C G T G A C G C T A C A T G A T C G T G A C C G A T A C T G T A G C G T A C G T C A | Tlx?(NR)/NPC-H3K4me1-ChIP-Seq(GSE16256)/Homer | 1e-20 | -4.803e+01 | 0.0000 | 407.0 | 6.42% | 1669.8 | 3.91% | motif file (matrix) | svg |
| 28 | A T G C T C G A A G T C A G C T A C G T G T A C A G T C G C T A C T A G C A T G G T C A C T G A T C A G A G T C | Stat3+il21(Stat)/CD4-Stat3-ChIP-Seq(GSE19198)/Homer | 1e-19 | -4.507e+01 | 0.0000 | 836.0 | 13.19% | 4112.2 | 9.62% | motif file (matrix) | svg |
| 29 | C G T A C G A T G A C T G A C T T G A C C T G A A T G C C T G A T A G C A G T C A C G T T C G A C A T G T A C G G A C T A T C G G A C T A C G T C T G A T C G A C G T A | Brachyury(T-box)/Mesoendoderm-Brachyury-ChIP-exo(GSE54963)/Homer | 1e-18 | -4.247e+01 | 0.0000 | 402.0 | 6.34% | 1700.6 | 3.98% | motif file (matrix) | svg |
| 30 | G A C T C A G T G A T C G A T C A C G T G A T C C T G A T A C G C G T A G T C A | STAT6(Stat)/Macrophage-Stat6-ChIP-Seq(GSE38377)/Homer | 1e-18 | -4.176e+01 | 0.0000 | 814.0 | 12.85% | 4039.0 | 9.45% | motif file (matrix) | svg |
| 31 | A G T C G A C T C A G T G T A C A G T C A T C G T C A G A C T G G T C A C G T A | Stat3(Stat)/mES-Stat3-ChIP-Seq(GSE11431)/Homer | 1e-18 | -4.173e+01 | 0.0000 | 569.0 | 8.98% | 2628.4 | 6.15% | motif file (matrix) | svg |
| 32 | C T G A A G C T A C G T A C G T A G T C G A C T G A C T C T G A C T G A C T A G C G T A C G T A | STAT6(Stat)/CD4-Stat6-ChIP-Seq(GSE22104)/Homer | 1e-16 | -3.774e+01 | 0.0000 | 810.0 | 12.78% | 4085.0 | 9.56% | motif file (matrix) | svg |
| 33 | C T G A A C G T A C G T A C G T A G T C G A C T C G A T C T G A A C T G C G T A C G T A T C G A | STAT5(Stat)/mCD4+-Stat5-ChIP-Seq(GSE12346)/Homer | 1e-14 | -3.308e+01 | 0.0000 | 537.0 | 8.47% | 2571.8 | 6.02% | motif file (matrix) | svg |
| 34 | T C A G A T C G G A C T A C T G G A C T C A G T C T A G C G T A G T A C C G T A C T A G A T C G | Tbx20(T-box)/Heart-Tbx20-ChIP-Seq(GSE29636)/Homer | 1e-13 | -3.108e+01 | 0.0000 | 252.0 | 3.98% | 1024.2 | 2.40% | motif file (matrix) | svg |
| 35 | C T G A T C A G G T A C G C T A A C T G T G A C G C A T C A T G | SCL(bHLH)/HPC7-Scl-ChIP-Seq(GSE13511)/Homer | 1e-13 | -3.094e+01 | 0.0000 | 3734.0 | 58.92% | 23179.1 | 54.25% | motif file (matrix) | svg |
| 36 | T C G A T A G C G T C A A C T G A C T G C G T A C G T A C T A G A G C T T C A G | ERG(ETS)/VCaP-ERG-ChIP-Seq(GSE14097)/Homer | 1e-13 | -3.056e+01 | 0.0000 | 1562.0 | 24.65% | 8876.6 | 20.78% | motif file (matrix) | svg |
| 37 | G A C T C G A T T C A G G A T C G A C T A G C T A G C T A G T C G A T C C G T A C T A G C T A G T C G A T C G A C T G A | Bcl6(Zf)/Liver-Bcl6-ChIP-Seq(GSE31578)/Homer | 1e-13 | -2.997e+01 | 0.0000 | 1659.0 | 26.18% | 9509.5 | 22.26% | motif file (matrix) | svg |
| 38 | T A G C T C A G C A T G G C A T A G C T C G A T A T G C C G T A C G T A G T C A | CHR(?)/Hela-CellCycle-Expression/Homer | 1e-12 | -2.835e+01 | 0.0000 | 1027.0 | 16.21% | 5590.7 | 13.08% | motif file (matrix) | svg |
| 39 | A G C T C T A G T G A C C G T A A C G T C G A T A G T C A G T C C T G A C A T G | TEAD3(TEA)/HepG2-TEAD3-ChIP-Seq(Encode)/Homer | 1e-11 | -2.757e+01 | 0.0000 | 1439.0 | 22.71% | 8183.2 | 19.15% | motif file (matrix) | svg |
| 40 | T C G A C T G A T A G C T G A C T C A G T C A G C G T A C G T A T C A G A G C T | ETV1(ETS)/GIST48-ETV1-ChIP-Seq(GSE22441)/Homer | 1e-11 | -2.734e+01 | 0.0000 | 1240.0 | 19.57% | 6938.3 | 16.24% | motif file (matrix) | svg |
| 41 | C A G T T C A G A G C T G A C T A C G T A G T C G A T C G A C T C T G A A C T G G A T C C G T A C T G A A G T C G T A C | Rfx6(HTH)/Min6b1-Rfx6.HA-ChIP-Seq(GSE62844)/Homer | 1e-11 | -2.545e+01 | 0.0000 | 962.0 | 15.18% | 5261.6 | 12.31% | motif file (matrix) | svg |
| 42 | T C G A T A G C T G C A A C T G A C T G C G T A C G T A C T A G G A C T T A C G | ETS1(ETS)/Jurkat-ETS1-ChIP-Seq(GSE17954)/Homer | 1e-11 | -2.541e+01 | 0.0000 | 987.0 | 15.58% | 5416.5 | 12.68% | motif file (matrix) | svg |
| 43 | C T A G T C G A C G A T C T A G G C A T C A G T C T A G G A T C C G T A G T C A | CEBP:AP1(bZIP)/ThioMac-CEBPb-ChIP-Seq(GSE21512)/Homer | 1e-10 | -2.465e+01 | 0.0000 | 968.0 | 15.28% | 5318.6 | 12.45% | motif file (matrix) | svg |
| 44 | T A G C G T A C C G T A C T A G A C T G T G C A C G T A A T G C C G T A A T C G | AR-halfsite(NR)/LNCaP-AR-ChIP-Seq(GSE27824)/Homer | 1e-10 | -2.394e+01 | 0.0000 | 3430.0 | 54.13% | 21380.5 | 50.04% | motif file (matrix) | svg |
| 45 | C G T A T A G C T A G C T G C A A C T G C T A G C G T A C G T A T C A G G A C T | EHF(ETS)/LoVo-EHF-ChIP-Seq(GSE49402)/Homer | 1e-10 | -2.345e+01 | 0.0000 | 1381.0 | 21.79% | 7939.2 | 18.58% | motif file (matrix) | svg |
| 46 | A G C T A G T C A T G C A G C T A C G T C G T A A C G T A G T C C G A T A T G C | Gata2(Zf)/K562-GATA2-ChIP-Seq(GSE18829)/Homer | 1e-10 | -2.324e+01 | 0.0000 | 876.0 | 13.82% | 4787.7 | 11.21% | motif file (matrix) | svg |
| 47 | T C G A T G A C G T A C C G T A C A G T T G A C A C G T A C T G A G C T A G C T | NeuroG2(bHLH)/Fibroblast-NeuroG2-ChIP-Seq(GSE75910)/Homer | 1e-9 | -2.281e+01 | 0.0000 | 1527.0 | 24.10% | 8889.5 | 20.81% | motif file (matrix) | svg |
| 48 | C T G A T A G C T G A C T C A G C T A G G T C A C G T A T C A G A G C T T C A G | ETV4(ETS)/HepG2-ETV4-ChIP-Seq(ENCODE)/Homer | 1e-9 | -2.273e+01 | 0.0000 | 865.0 | 13.65% | 4732.6 | 11.08% | motif file (matrix) | svg |
| 49 | T C A G T A C G T A G C A C G T A C T G C G A T A G T C C G T A T A C G A G T C | Meis1(Homeobox)/MastCells-Meis1-ChIP-Seq(GSE48085)/Homer | 1e-9 | -2.252e+01 | 0.0000 | 1541.0 | 24.32% | 8988.7 | 21.04% | motif file (matrix) | svg |
| 50 | A T G C A G T C C T G A A G T C C G A T A C G T A G T C A G T C A C G T A T C G G A C T A C G T | Etv2(ETS)/ES-ER71-ChIP-Seq(GSE59402)/Homer | 1e-9 | -2.196e+01 | 0.0000 | 913.0 | 14.41% | 5048.7 | 11.82% | motif file (matrix) | svg |
| 51 | T A G C G C T A A C T G C G T A A C G T C G T A C G T A T A C G T C A G T C G A | Gata1(Zf)/K562-GATA1-ChIP-Seq(GSE18829)/Homer | 1e-9 | -2.166e+01 | 0.0000 | 769.0 | 12.14% | 4170.2 | 9.76% | motif file (matrix) | svg |
| 52 | T G C A A G C T A C G T C T A G G A T C C T A G G A T C G T C A C T G A A G T C | CEBP(bZIP)/ThioMac-CEBPb-ChIP-Seq(GSE21512)/Homer | 1e-9 | -2.149e+01 | 0.0000 | 859.0 | 13.56% | 4727.5 | 11.06% | motif file (matrix) | svg |
| 53 | A G C T A T G C G A C T G C A T C G T A A G C T G T A C C G A T A T C G A G T C | Gata6(Zf)/HUG1N-GATA6-ChIP-Seq(GSE51936)/Homer | 1e-9 | -2.109e+01 | 0.0000 | 1259.0 | 19.87% | 7240.3 | 16.95% | motif file (matrix) | svg |
| 54 | T A G C T A G C G C A T C A T G A C T G G C T A C G T A A C G T A C T G G A T C | TEAD4(TEA)/Tropoblast-Tead4-ChIP-Seq(GSE37350)/Homer | 1e-8 | -1.960e+01 | 0.0000 | 949.0 | 14.98% | 5337.1 | 12.49% | motif file (matrix) | svg |
| 55 | A G C T G C A T A C T G A C G T A G T C A C G T C T A G T A C G | Smad3(MAD)/NPC-Smad3-ChIP-Seq(GSE36673)/Homer | 1e-8 | -1.939e+01 | 0.0000 | 2607.0 | 41.14% | 16064.4 | 37.60% | motif file (matrix) | svg |
| 56 | C T A G T C G A T G A C A G T C C G T A A C T G G T A C A C G T A C T G A C T G | BHLHA15(bHLH)/NIH3T3-BHLHB8.HA-ChIP-Seq(GSE119782)/Homer | 1e-8 | -1.910e+01 | 0.0000 | 1322.0 | 20.86% | 7706.6 | 18.04% | motif file (matrix) | svg |
| 57 | A T G C C T G A G A C T A C G T A C G T G T A C G A T C C G A T C T A G C A T G C G T A C G T A C T G A G A C T | STAT1(Stat)/HelaS3-STAT1-ChIP-Seq(GSE12782)/Homer | 1e-8 | -1.902e+01 | 0.0000 | 437.0 | 6.90% | 2230.1 | 5.22% | motif file (matrix) | svg |
| 58 | A G T C C T G A A G T C C G A T C A G T G A T C A T G C A C T G A T C G G A C T | Fli1(ETS)/CD8-FLI-ChIP-Seq(GSE20898)/Homer | 1e-8 | -1.900e+01 | 0.0000 | 939.0 | 14.82% | 5292.2 | 12.39% | motif file (matrix) | svg |
| 59 | T G C A A G C T C A T G C G T A A G C T A C T G G A T C G T C A C G T A A G C T | Atf4(bZIP)/MEF-Atf4-ChIP-Seq(GSE35681)/Homer | 1e-8 | -1.888e+01 | 0.0000 | 382.0 | 6.03% | 1910.4 | 4.47% | motif file (matrix) | svg |
| 60 | T C A G A C G T T C G A T A G C A G T C C G T A A C T G G T A C A C G T A C T G A T C G A G T C | Atoh1(bHLH)/Cerebellum-Atoh1-ChIP-Seq(GSE22111)/Homer | 1e-8 | -1.869e+01 | 0.0000 | 958.0 | 15.12% | 5420.4 | 12.69% | motif file (matrix) | svg |
| 61 | T A C G A T G C G C T A A C T G C G T A A C G T C G T A C T G A T A C G T C G A | Gata4(Zf)/Heart-Gata4-ChIP-Seq(GSE35151)/Homer | 1e-8 | -1.863e+01 | 0.0000 | 1319.0 | 20.81% | 7703.9 | 18.03% | motif file (matrix) | svg |
| 62 | C T G A A T G C C G T A A C G T A G T C A G T C A C G T A C T G A T C G G C A T | SPDEF(ETS)/VCaP-SPDEF-ChIP-Seq(SRA014231)/Homer | 1e-8 | -1.858e+01 | 0.0000 | 991.0 | 15.64% | 5630.6 | 13.18% | motif file (matrix) | svg |
| 63 | T G A C C T G A C T A G C T G A C G T A A G T C C T G A A C G T G C A T T A G C G C A T A T C G G A C T G A C T G A T C | GRE(NR),IR3/RAW264.7-GRE-ChIP-Seq(Unpublished)/Homer | 1e-7 | -1.740e+01 | 0.0000 | 281.0 | 4.43% | 1352.6 | 3.17% | motif file (matrix) | svg |
| 64 | C G A T C T G A C G T A C A G T A G T C G A T C G A T C A C T G | Pitx1(Homeobox)/Chicken-Pitx1-ChIP-Seq(GSE38910)/Homer | 1e-6 | -1.567e+01 | 0.0000 | 4397.0 | 69.39% | 28357.0 | 66.37% | motif file (matrix) | svg |
| 65 | T C G A A G C T A C G T A C G T A G T C A G T C A C G T A T C G G A C T A T C G | EWS:ERG-fusion(ETS)/CADO\_ES1-EWS:ERG-ChIP-Seq(SRA014231)/Homer | 1e-6 | -1.552e+01 | 0.0000 | 882.0 | 13.92% | 5042.0 | 11.80% | motif file (matrix) | svg |
| 66 | A C G T T A C G G A T C A C T G A C G T C T A G A C T G A C T G G A T C C T A G C A T G C T A G | Egr2(Zf)/Thymocytes-Egr2-ChIP-Seq(GSE34254)/Homer | 1e-6 | -1.535e+01 | 0.0000 | 101.0 | 1.59% | 393.8 | 0.92% | motif file (matrix) | svg |
| 67 | T G A C A G T C C G T A A C T G G T A C A C G T A C T G A C G T G A C T G A T C | Twist2(bHLH)/Myoblast-Twist2.Ty1-ChIP-Seq(GSE127998)/Homer | 1e-6 | -1.529e+01 | 0.0000 | 1664.0 | 26.26% | 10054.6 | 23.53% | motif file (matrix) | svg |
| 68 | T G A C A G T C C T G A T G A C C G T A A C G T A C G T A G T C A G T C C G T A | TEAD1(TEAD)/HepG2-TEAD1-ChIP-Seq(Encode)/Homer | 1e-6 | -1.523e+01 | 0.0000 | 1168.0 | 18.43% | 6862.4 | 16.06% | motif file (matrix) | svg |
| 69 | C T G A T G A C T G A C C G T A A C G T T G A C A G C T C T A G A C G T G A C T | Olig2(bHLH)/Neuron-Olig2-ChIP-Seq(GSE30882)/Homer | 1e-6 | -1.500e+01 | 0.0000 | 1997.0 | 31.51% | 12240.4 | 28.65% | motif file (matrix) | svg |
| 70 | C G T A C T G A C T A G C G T A C G T A A G T C C G T A C A G T G C A T G T C A C G A T A C T G A C G T G C A T G A T C | PGR(NR)/EndoStromal-PGR-ChIP-Seq(GSE69539)/Homer | 1e-6 | -1.483e+01 | 0.0000 | 331.0 | 5.22% | 1687.8 | 3.95% | motif file (matrix) | svg |
| 71 | T A C G T G C A A G T C C G T A A C G T T G A C A C G T A C T G A C T G G C A T | TCF4(bHLH)/SHSY5Y-TCF4-ChIP-Seq(GSE96915)/Homer | 1e-6 | -1.465e+01 | 0.0000 | 1469.0 | 23.18% | 8818.2 | 20.64% | motif file (matrix) | svg |
| 72 | T C G A T C G A T A G C G T A C T C A G T A C G C G T A C G T A T C A G A G C T | GABPA(ETS)/Jurkat-GABPa-ChIP-Seq(GSE17954)/Homer | 1e-6 | -1.390e+01 | 0.0000 | 731.0 | 11.54% | 4148.4 | 9.71% | motif file (matrix) | svg |
| 73 | C G T A C A T G C A T G A C T G C T A G T C G A G C A T C G A T A G C T A G T C G A T C G T A C | NFkB-p65(RHD)/GM12787-p65-ChIP-Seq(GSE19485)/Homer | 1e-6 | -1.389e+01 | 0.0000 | 412.0 | 6.50% | 2189.5 | 5.12% | motif file (matrix) | svg |
| 74 | C G T A T G A C T A G C T G C A A C T G A C T G C G T A C G T A T C A G G A C T | ELF3(ETS)/PDAC-ELF3-ChIP-Seq(GSE64557)/Homer | 1e-5 | -1.346e+01 | 0.0000 | 802.0 | 12.66% | 4608.0 | 10.78% | motif file (matrix) | svg |
| 75 | A T G C G A C T A C T G C A G T G A T C A C G T T A C G T A C G | Smad2(MAD)/ES-SMAD2-ChIP-Seq(GSE29422)/Homer | 1e-5 | -1.307e+01 | 0.0000 | 1302.0 | 20.55% | 7807.7 | 18.27% | motif file (matrix) | svg |
| 76 | C T G A A G T C C G A T A G C T A T G C G T A C A C G T A T C G C A G T G C A T | Elf4(ETS)/BMDM-Elf4-ChIP-Seq(GSE88699)/Homer | 1e-5 | -1.289e+01 | 0.0000 | 901.0 | 14.22% | 5251.1 | 12.29% | motif file (matrix) | svg |
| 77 | T A C G A T C G T A G C G A T C A C T G A C G T A G T C A C G T C T A G A T C G | Smad4(MAD)/ESC-SMAD4-ChIP-Seq(GSE29422)/Homer | 1e-5 | -1.280e+01 | 0.0000 | 1292.0 | 20.39% | 7754.2 | 18.15% | motif file (matrix) | svg |
| 78 | G A C T C T A G C T A G A G T C T G C A A C T G A C G T A C G T C T A G T C A G | AMYB(HTH)/Testes-AMYB-ChIP-Seq(GSE44588)/Homer | 1e-5 | -1.253e+01 | 0.0000 | 1459.0 | 23.02% | 8846.5 | 20.70% | motif file (matrix) | svg |
| 79 | T C A G T G A C G T A C C G T A A C G T T G A C A C G T T C A G A G C T G A C T | NeuroD1(bHLH)/Islet-NeuroD1-ChIP-Seq(GSE30298)/Homer | 1e-5 | -1.214e+01 | 0.0000 | 723.0 | 11.41% | 4155.7 | 9.73% | motif file (matrix) | svg |
| 80 | C G T A A C G T A C G T A C G T A C G T A G T C A G T C C T G A A G C T A G C T | NFAT(RHD)/Jurkat-NFATC1-ChIP-Seq(Jolma\_et\_al.)/Homer | 1e-5 | -1.209e+01 | 0.0000 | 1190.0 | 18.78% | 7128.9 | 16.68% | motif file (matrix) | svg |
| 81 | A T G C G A T C G A C T A G C T C G A T C G A T G T C A C G A T T C G A A T C G T A G C T A G C | TATA-Box(TBP)/Promoter/Homer | 1e-4 | -1.097e+01 | 0.0001 | 1645.0 | 25.96% | 10133.0 | 23.72% | motif file (matrix) | svg |
| 82 | G C T A A T C G G C T A G A C T G C T A T C G A T A G C T C G A | GATA3(Zf)/iTreg-Gata3-ChIP-Seq(GSE20898)/Homer | 1e-4 | -1.085e+01 | 0.0001 | 1974.0 | 31.15% | 12300.0 | 28.79% | motif file (matrix) | svg |
| 83 | T A C G C T G A C T A G T C G A C G T A A G T C C T G A A T C G G C A T T A G C G A C T A C T G A C G T A G C T A G T C G A C T | GRE(NR),IR3/A549-GR-ChIP-Seq(GSE32465)/Homer | 1e-4 | -1.072e+01 | 0.0001 | 145.0 | 2.29% | 683.6 | 1.60% | motif file (matrix) | svg |
| 84 | T G C A C G T A G T C A A G C T A G T C G C T A T A G C C G A T C T A G G A T C | Gfi1b(Zf)/HPC7-Gfi1b-ChIP-Seq(GSE22178)/Homer | 1e-4 | -1.046e+01 | 0.0002 | 735.0 | 11.60% | 4290.2 | 10.04% | motif file (matrix) | svg |
| 85 | A G T C G C A T C G T A C G T A G T A C A C G T A C T G G A T C G A T C T C G A | BMYB(HTH)/Hela-BMYB-ChIP-Seq(GSE27030)/Homer | 1e-4 | -1.034e+01 | 0.0002 | 1481.0 | 23.37% | 9095.9 | 21.29% | motif file (matrix) | svg |
| 86 | T C G A A C T G A C T G C G T A C G T A T C G A A G T C C T G A A T C G G T A C G C A T C A T G | ETS:E-box(ETS,bHLH)/HPC7-Scl-ChIP-Seq(GSE22178)/Homer | 1e-4 | -1.032e+01 | 0.0002 | 102.0 | 1.61% | 450.2 | 1.05% | motif file (matrix) | svg |
| 87 | T A C G C T G A T C G A C G A T C T A G C T A G T C G A C T G A T C G A T C G A C G T A T C G A G C A T C A T G C G T A T A C G G C A T T G A C C G T A A G C T | NFAT:AP1(RHD,bZIP)/Jurkat-NFATC1-ChIP-Seq(Jolma\_et\_al.)/Homer | 1e-4 | -9.733e+00 | 0.0003 | 217.0 | 3.42% | 1116.2 | 2.61% | motif file (matrix) | svg |
| 88 | T G C A C T G A A G T C G T C A A C T G A C T G C G T A C G T A C T G A A G C T | EWS:FLI1-fusion(ETS)/SK\_N\_MC-EWS:FLI1-ChIP-Seq(SRA014231)/Homer | 1e-4 | -9.226e+00 | 0.0005 | 530.0 | 8.36% | 3044.8 | 7.13% | motif file (matrix) | svg |
| 89 | C T A G C T A G A G T C T C A G A C T G A C G T A C G T C T G A | MYB(HTH)/ERMYB-Myb-ChIPSeq(GSE22095)/Homer | 1e-3 | -9.124e+00 | 0.0005 | 1656.0 | 26.13% | 10306.9 | 24.12% | motif file (matrix) | svg |
| 90 | C T A G C G T A G T C A C G T A A G T C G A T C A G C T C T A G C G T A A C G T G T C A G A T C | Six2(Homeobox)/NephronProgenitor-Six2-ChIP-Seq(GSE39837)/Homer | 1e-3 | -8.894e+00 | 0.0007 | 1171.0 | 18.48% | 7154.1 | 16.74% | motif file (matrix) | svg |
| 91 | G A T C G T A C C G A T A C T G A C T G C G T A C G T A A C G T A C T G G A T C | TEAD(TEA)/Fibroblast-PU.1-ChIP-Seq(Unpublished)/Homer | 1e-3 | -8.410e+00 | 0.0011 | 853.0 | 13.46% | 5126.7 | 12.00% | motif file (matrix) | svg |
| 92 | C G T A A C T G C G T A A C G T C G T A C T G A T C A G T G C A A G C T T G A C | TRPS1(Zf)/MCF7-TRPS1-ChIP-Seq(GSE107013)/Homer | 1e-3 | -8.362e+00 | 0.0011 | 2628.0 | 41.47% | 16797.0 | 39.31% | motif file (matrix) | svg |
| 93 | C G A T T G C A T G C A G A T C C G T A A C T G T G A C G A C T C A T G A C T G | Tcf21(bHLH)/ArterySmoothMuscle-Tcf21-ChIP-Seq(GSE61369)/Homer | 1e-3 | -7.896e+00 | 0.0018 | 721.0 | 11.38% | 4304.4 | 10.07% | motif file (matrix) | svg |
| 94 | A T C G G A T C G C A T C G T A G T C A A C G T A T G C A G T C | CRX(Homeobox)/Retina-Crx-ChIP-Seq(GSE20012)/Homer | 1e-3 | -7.757e+00 | 0.0020 | 2660.0 | 41.98% | 17053.0 | 39.91% | motif file (matrix) | svg |
| 95 | T A G C G C A T A G T C G A T C A T G C G A C T C T A G A C T G A C T G C T G A A C T G C T A G A G T C T G A C C G A T | GLIS3(Zf)/Thyroid-Glis3.GFP-ChIP-Seq(GSE103297)/Homer | 1e-3 | -7.408e+00 | 0.0028 | 676.0 | 10.67% | 4038.1 | 9.45% | motif file (matrix) | svg |
| 96 | T G A C C T A G T C A G G T C A C G T A T C A G C G A T T C A G T C G A T G C A C T G A T A G C | PU.1-IRF(ETS:IRF)/Bcell-PU.1-ChIP-Seq(GSE21512)/Homer | 1e-3 | -7.307e+00 | 0.0031 | 1539.0 | 24.29% | 9649.0 | 22.58% | motif file (matrix) | svg |
| 97 | G C A T A T C G C A T G G T A C G C T A A G T C T C A G T G A C G T C A T G C A | Arnt:Ahr(bHLH)/MCF7-Arnt-ChIP-Seq(Lo\_et\_al.)/Homer | 1e-3 | -7.257e+00 | 0.0032 | 486.0 | 7.67% | 2838.6 | 6.64% | motif file (matrix) | svg |
| 98 | C A T G A G T C C T G A T G A C A T C G G A C T G T C A A G T C T A G C G A T C | HIF2a(bHLH)/785\_O-HIF2a-ChIP-Seq(GSE34871)/Homer | 1e-3 | -6.974e+00 | 0.0042 | 236.0 | 3.72% | 1293.9 | 3.03% | motif file (matrix) | svg |
| 99 | A C T G C A T G G C T A T C G A G C T A A G C T A G C T G T A C A G T C T G A C | NFkB-p65-Rel(RHD)/ThioMac-LPS-Expression(GSE23622)/Homer | 1e-2 | -6.907e+00 | 0.0044 | 56.0 | 0.88% | 241.3 | 0.56% | motif file (matrix) | svg |
| 100 | A C T G A C G T C A T G A T C G A T C G T G A C A C T G A T C G A T C G T G C A C T G A C G T A | E2F3(E2F)/MEF-E2F3-ChIP-Seq(GSE71376)/Homer | 1e-2 | -6.836e+00 | 0.0047 | 198.0 | 3.12% | 1066.6 | 2.50% | motif file (matrix) | svg |
| 101 | A T G C A G T C G T A C A G C T T C G A C T A G G A T C C T G A G T C A A G T C G C T A T C A G | Rfx5(HTH)/GM12878-Rfx5-ChIP-Seq(GSE31477)/Homer | 1e-2 | -6.477e+00 | 0.0067 | 380.0 | 6.00% | 2200.6 | 5.15% | motif file (matrix) | svg |
| 102 | G C T A A G T C T A C G T G C A A T C G T C A G G C T A T C G A T C A G A G C T | ELF5(ETS)/T47D-ELF5-ChIP-Seq(GSE30407)/Homer | 1e-2 | -6.417e+00 | 0.0070 | 724.0 | 11.42% | 4392.4 | 10.28% | motif file (matrix) | svg |
| 103 | C T G A T C A G A G T C C G T A A T C G A T G C C G A T A C T G A G T C G A C T A T C G A G T C | MyoD(bHLH)/Myotube-MyoD-ChIP-Seq(GSE21614)/Homer | 1e-2 | -6.158e+00 | 0.0090 | 515.0 | 8.13% | 3066.4 | 7.18% | motif file (matrix) | svg |
| 104 | T G C A T C G A T C G A A C G T C A T G C G T A A G T C T C A G A C G T G T A C C G T A A G C T | CREB5(bZIP)/LNCaP-CREB5.V5-ChIP-Seq(GSE137775)/Homer | 1e-2 | -5.972e+00 | 0.0108 | 375.0 | 5.92% | 2187.8 | 5.12% | motif file (matrix) | svg |
| 105 | G A C T C G A T C T G A G T C A G A C T C G A T T C G A C G T A G C T A G C T A T G A C G T A C C G T A A C T G T G C A C G A T A C T G A C G T | Pitx1:Ebox(Homeobox,bHLH)/Hindlimb-Pitx1-ChIP-Seq(GSE41591)/Homer | 1e-2 | -5.776e+00 | 0.0130 | 275.0 | 4.34% | 1568.6 | 3.67% | motif file (matrix) | svg |
| 106 | T C A G A G C T A T G C C G T A A G T C T C A G A C G T A T C G T C G A A G T C G A T C T G A C | TFE3(bHLH)/MEF-TFE3-ChIP-Seq(GSE75757)/Homer | 1e-2 | -5.751e+00 | 0.0132 | 70.0 | 1.10% | 333.7 | 0.78% | motif file (matrix) | svg |
| 107 | A G T C G A C T A C T G G A T C G T A C C G T A T G A C A G T C C G A T A G C T A C G T A C G T C T A G G A C T C T G A | ZNF7(Zf)/HepG2-ZNF7.Flag-ChIP-Seq(Encode)/Homer | 1e-2 | -5.592e+00 | 0.0153 | 665.0 | 10.49% | 4054.3 | 9.49% | motif file (matrix) | svg |
| 108 | T G C A C T G A A T G C G T C A A C T G A C T G C G T A C G T A C T A G A G C T | Ets1-distal(ETS)/CD4+-PolII-ChIP-Seq(Barski\_et\_al.)/Homer | 1e-2 | -5.144e+00 | 0.0238 | 293.0 | 4.62% | 1702.9 | 3.99% | motif file (matrix) | svg |
| 109 | A G T C G T A C A G C T C T A G A G T C C G A T A C T G C G T A A C T G G T C A | Zic(Zf)/Cerebellum-ZIC1.2-ChIP-Seq(GSE60731)/Homer | 1e-2 | -5.135e+00 | 0.0238 | 582.0 | 9.18% | 3543.0 | 8.29% | motif file (matrix) | svg |
| 110 | G A C T A G C T G T A C G A C T C T G A A C T G G T C A C T G A A T G C T A C G G A C T A C G T A G T C G A C T C T G A | HRE(HSF)/Striatum-HSF1-ChIP-Seq(GSE38000)/Homer | 1e-2 | -5.083e+00 | 0.0248 | 262.0 | 4.13% | 1510.4 | 3.53% | motif file (matrix) | svg |
| 111 | T C G A G C A T A C G T C T A G G T A C T C G A G C A T T G A C T C G A A C G T | Chop(bZIP)/MEF-Chop-ChIP-Seq(GSE35681)/Homer | 1e-2 | -4.951e+00 | 0.0280 | 256.0 | 4.04% | 1477.4 | 3.46% | motif file (matrix) | svg |
| 112 | T A C G T A C G G T A C A T C G A C T G T A C G T C G A C T G A T C G A A T C G | E2F6(E2F)/Hela-E2F6-ChIP-Seq(GSE31477)/Homer | 1e-2 | -4.902e+00 | 0.0292 | 167.0 | 2.64% | 927.8 | 2.17% | motif file (matrix) | svg |
